# Kinetic and Kinematic Characteristics of Proficient and Non-Proficient 2-Point and 3-Point Basketball Shooters

**DOI:** 10.3390/sports10010002

**Published:** 2021-12-22

**Authors:** Dimitrije Cabarkapa, Andrew C. Fry, Damjana V. Cabarkapa, Chloe A. Myers, Grant T. Jones, Michael A. Deane

**Affiliations:** Jayhawk Athletic Performance Laboratory—Wu Tsai Human Performance Alliance, University of Kansas, Lawrence, KS 66045, USA; acfry@ku.edu (A.C.F.); d927c184@ku.edu (D.V.C.); chloe.myers@burrell.edu (C.A.M.); gtjohnes02@ku.edu (G.T.J.); potsdam24@aol.com (M.A.D.)

**Keywords:** biomechanics, sport science, performance, team sports, shooting

## Abstract

The purpose of this study was to examine kinetic and kinematic characteristics of various types of 2-point and 3-point basketball shooting approaches and determine which variables have the greatest contribution in discriminating proficient (PRO) from non-proficient (N-PRO) shooters. While standing on a force plate, twenty-nine recreationally active males performed a total of 1740 shots by utilizing stationary and step-in shooting approaches. Two high-definition cameras were used to simultaneously capture kinematic parameters of shooting motions. The type of shooting approach showed as a non-influential factor. During the preparatory phase of the shooting motion, PRO 2-point shooters demonstrated higher elbow and basketball height placements, greater flexion in the shoulder and elbow joints while attaining greater release and entry ball angles during the release phase. PRO 3-point shooters demonstrated greater elbow flexion, higher basketball placement, and less hip flexion during the preparatory phase while attaining greater heel, release, and trajectory heights during the release phase. When entered into a full-model discriminant function analysis, elbow angle, elbow height, and release angle variables correctly classified PRO from N-PRO 2-point shooters in 62.1% of cases and hip angle, heel height, and elbow angle variables correctly classified PRO from N-PRO 3-point shooters in 81.6% of cases.

## 1. Introduction

Basketball is one of the most popular sports played on various levels of international competition. The fast pace of play resulting in scoring opportunities every few ball possessions, rapid changes in score, and highly athletic motions make this sport appealing to a large audience. While performance indicators such as rebounds, assists, personal fouls, and turnovers may impact the desired game outcome, the only way for teams to score points is by putting the ball through the hoop. Besides attempting free-throw shots as an uncontested scoring opportunity, this can be achieved by performing different types of 2-point, and 3-point shooting motions. Previous research has indicated that winning teams had significantly greater 2-point and 3-point field goal percentages, suggesting better offensive discipline and overall offensive organization [1,2,3]. Moreover, successful 2-point and 3-point field goal performance was one of the factors with the greatest discriminating power in distinguishing between winning and losing teams on an elite level of professional basketball competition [3]. Hence, considering the impact on securing a victorious game outcome, a detailed analysis of biomechanical parameters of these types of shooting motions is crucial for obtaining a better insight on proper shooting technique, and assuring further improvements in athletes’ shooting performances.

Based on previous biomechanical research, some of the vital cues given to athletes while teaching or coaching a basketball jump-shooting motion (i.e., 2-point and 3-point shots) should be focused on addressing optimal height of the release, angle of release, coordination between upper and lower extremities, and appropriate body alignment and vertical jump displacement [4]. When examining mid-range jump-shooting performance, more successful shooters demonstrated greater release angle, smaller elbow angle, greater spin, and less lateral elbow deviation when compared to a vertical imaginary axis [5]. Similarly, in a recently published study, forearm positioning was found to be a key kinematic variable capable of distinguishing between proficient and non-proficient free-throw shooters [6]. To couple with previously mentioned findings Inaba et al. [7] found that players with superior mid-range jump-shot performance had greater release height, lower release velocity, and a larger margin of error for the release speed. The authors also suggested that an increase in backspin rate may help with maintaining optimal release parameters such as speed and angle of the release, especially at lower release heights [7]. To apply appropriate backspin for the proper execution of the jump shooting motion, a large vertical fingertip acceleration relative to the shoulder is needed [8]. Based on a numerical simulation model, it has been determined that the vertical component of release velocity is mainly attributed to rotation in the shoulder joint, while wrist and elbow actions are responsible for horizontal release velocity and backspin [9]. 

Unlike the regulations for the free-throw shooting motion that remain consistent across all levels of basketball competition, the distance from which the jump-shooting motion is being performed have a considerable impact on its biomechanical parameters [10,11,12]. Okazaki & Rodacki [10] have found that shooting accuracy, release height, and release angle (only for close to mid-range shots) were inversely related to an increase in jump-shot shooting distance. Likewise, increased ball release speed caused by the greater center of body mass and elbow extension velocities, and decrease in time from take-off to release were additional biomechanical adjustments made to offset the impact of an increase in shooting distance [11]. While multiple adjustments in shooting technique in fact occur, their specificity and magnitude may be dependent on a playing position that is primarily determined by the athlete’s anthropometric characteristics [12]. With release angle decreasing uniformly across all playing positions as players attempt long-range shots, a significant increase in release speed was noted only for guards [13]. Centers had a lower angle of ball entry when compared to forwards and guards [14]. Also, changes in kinematic parameters were more consistent in guards when compared to forwards and centers, which may be mainly attributed to on-court playing demands and frequency of practice sessions tailored towards improvements in long-range jump-shooting performance [13]. Moreover, considering that recent changes in the 3-point line regulation distance led to a significant decrease in the number of made and attempted long-range jump-shots [15], we may assume that this may require adjustments to biomechanical parameters of jump-shot shooting motions to a greater extent.

As with any type of body motion, performing an optimal jump-shot requires multi-segmental body contributions. The majority of previous research has been focused on addressing biomechanical parameters around the timepoint of the ball release and what changes occur with an increase in shooting distance, leaving the preparatory phase of this type of shooting motion vastly unexplored. In addition, there is a lack of scientific literature addressing differences in kinetic and kinematic characteristics between proficient and non-proficient jump-shooters. In a recently conducted study, Cabarkapa et al. [16] found that proficient free-throw shooters demonstrated greater flexion in the knee, hip, and ankle joints which ultimately led to a greater number of made shots. Proficient shooters also attained greater elbow height during the preparatory phase of the shooting motion suggesting that the ball was positioned at the upper-chest area commonly defined as a “shot pocket” [16]. Therefore, the purpose of this study was to (a) examine differences in kinetic and kinematic characteristics between proficient and non-proficient 2-point and 3-point shooters, (b) examine the influence of different types of shooting approaches on changes in kinetic and kinematic characteristics of 2-point and 3-point shooting motions, and (c) determine which kinetic and kinematic variables have the greatest contribution in discriminating proficient from non-proficient 2-point and 3-point shooters. 

## 2. Materials and Methods

### 2.1. Participants

Twenty-nine recreationally active college-aged males ($\bar{x}$ ± SD; height = 182.6 ± 9.1 cm; body mass = 84.1 ± 15.4 kg; age = 22.6 ± 4.1 years; basketball playing experience = 7.3 ± 2.8 years) participating in at least 150 min of moderate physical activity per week volunteered to participate in the present study. All participants previously competed on high school level of basketball competition and were right-hand dominant shooters. To assure optimal assessment of biomechanical parameters of various types of basketball shooting motions, participants with current and previous musculoskeletal injuries that could potentially impact a full joint range of motion were excluded from participation. All testing procedures performed in this study were previously approved by the University’s Institutional Review Board and all participants signed the informed consent document. 

### 2.2. Procedures

Upon arrival to the laboratory, participants performed a standardized warm-up procedure consisting of a set of dynamic stretching exercises (e.g., butt-kicks, quad pulls, lateral lunges, A-skips, walking quad stretch). Each participant was allowed to take 5–10 practice shots from self-selected distances before the start of the testing procedures. While standing on a uni-dimensional force plate (0.91 m × 2.44 m, RoughDeck, Rice Lake, WI, USA) each participant performed 60 shots: 10 stationary 2-point and 10 stationary 3-point shots, 10 step-in 2-point and 10 step-in 3-point shots where subjects took a penultimate step with the left foot to establish the initial ground contact followed by the right foot as a push-off action, and 10 step-in 2-point and 10 step-in 3-point shots in reverse order with the right foot serving as a penultimate step. The total number of shots attempted across all participants was 1740. A data acquisition system (BioPac Systems Inc., Goleta, CA, USA) sampling at 1000 Hz was used to collect ground reaction forces from which kinetic variables of interest were computed. Simultaneously, two high-definition cameras (Canon PowerShot SX530, Canon Inc., Tokyo, Japan) recording at 30 fps were used to capture kinematic parameters of different types of shooting motion examined in this study. The first camera was positioned 10 m away perpendicular to the participant shooting location (2-point or 3-point) to capture a sagittal view of kinematic variables during preparatory and release phases of the shooting motions. The second camera was positioned 20 m away perpendicular to the mid-point between the specific shooting location and the rim to capture a sagittal view of kinematic parameters of the basketball trajectory. Video analysis software (Kinovea, Version 0.8.27) was used to analyze the kinematic variables of interest. In order to eliminate a possible influence of fatigue, each shot and shooting approach were separated by 5–10 s and 1–2 min rest interval, respectively. Regardless of the basketball shooting approach, both 2-point and 3-point shots were attempted directly facing the basket from identical distances, 5.10 m and 6.75 m, respectively. No instruction regarding the shooting technique has been given to any of the participants throughout testing. A research assistant was present throughout the full testing procedure to help with rebounding and passing tasks. To eliminate any kind of possible distractions, participants individually performed all testing procedures. Basketball goal height (3.05 m) and size (0.75 m) corresponded to men’s basketball international regulations standards. Based on feedback from a panel of experts (i.e., former collegiate and professional basketball coaches and players), participants making ≥50% of their 2-point and ≥40% of their 3-point shots were classified as proficient (PRO; *n* = 13) and those making less than the previously indicated cutoff points were categorized as non-proficient (N-PRO; *n* = 16).

### 2.3. Variables

The kinetic and kinematic variables examined in the present study were selected based on the previously conducted scientific literature [4,5,6,7,9,12,17] and an extensive conversation with a panel of basketball coaches with 50+ years of coaching experience. 

Kinematic variables assessed at the initial concentric phase of the shooting motion were *knee angle* (internal angle between the thigh and shank), *hip angle* (internal angle between the torso and the thigh), *ankle angle* (relative angle between the shank and the ground), *elbow angle* (internal angle between the upper arm and forearm), *shoulder angle* (relative angle between the upper arm and torso), *elbow height* (perpendicular distance between the olecranon process and the ground divided by participant’s height), and *basketball height* (perpendicular distance between the center of the ball and the ground divided by participant’s height). 

Kinematic variables assessed at the time point of the ball release were *release angle* (relative angle between the fully extended upper limb and a line parallel to the ground), *release height* (perpendicular distance between the center of the basketball and the ground divided by the participant’s height), and *heel height* (perpendicular distance between the calcaneus and the ground). Also, kinematic variables pertaining to the path of the ball while in flight after being released by the shooter were *maximal trajectory height* (perpendicular distance from the center of the basketball to the ground at the highest trajectory point) and *entry angle* (relative angle at which basketball enters the rim).

Kinetic variables examined in the present investigation include *peak concentric force* (greatest ground reaction force recorded during the concentric phase of the shooting motion), *peak landing force* (greatest ground reaction force recorded during the landing phase of the shooting motion), *rate of force development* (slope between the timepoint when the ground reaction force reached the subject’s body weight and the peak concentric force), and *impulse* (calculated as an area under the curve above the subject’s body weight during the concentric phase of the shooting motion). 

### 2.4. Statistical Analysis

Descriptive statistics, means and standard deviations ($\bar{x}$ ± SD), were calculated for each dependent variable. A repeated measures MANOVA with Bonferroni adjustments was used to compare main effects and interactions between different types of shooting approaches (stationary, step-in left, and step-in right), distances (2-point and 3-point), and proficiencies (PRO and N-PRO). Follow-up ANOVAs were used to examine statistically significant main effects where needed. Pearson-product moment correlation coefficients (*r*) were used to inspect the relationships between the dependent variables. As a measure of relative variability, the coefficient of variation (CV) was calculated for each dependent variable. A full model discriminant function analysis was used to examine the magnitude of the relative contribution of the dependent variables and classify PRO from N-PRO shooters, separately for 2-point and 3-point shooting distances. Based on the shared variances and the sample size (*n* = 29), related variables were removed, resulting in three performance variables for each analysis. Statistical significance was set a priori to *p* < 0.05. All statistical analyses were completed with SPSS (Version 26.0; IBM Corp., Armonk, NY, USA). 

## 3. Results

The average shooting accuracy (%) for 2-point PRO and N-PRO shooters were 67.7 ± 12.4 and 39.2 ± 9.6, and for 3-point shooters 59.4 ± 14.4 and 28.2 ± 9.1, respectively. The MANOVA indicated a non-significant interaction between shooting proficiency, approach, and distance (F[32,294] = 0.194, *p* = 0.997, Λ = 0.959, *η*_p^2^_ = 0.021). Also, no interaction effects were found between shooting approach and distance (F[32,294] = 0.226, *p* = 0.998, Λ = 0.953, *η*_p^2^_ = 0.024), and shooting approach and proficiency (F[32,294] = 0.183, *p* = 0.998, Λ = 0.961, *η*_p^2^_ = 0.020). Thus, the influence of the shooting approach on kinetic and kinematic parameters of 2-point and 3-point shooting motions has been excluded from further data analysis as a non-influential factor. A significant main effect was found for shooting distance (F[16,147] = 22.286, *p* = < 0.001, Λ = 0.292, *η*_p^2^_ = 0.708) as well as the interaction between shooting distance and proficiency (F[16,147] = 2.836, *p* = < 0.001, Λ = 0.764, *η*_p^2^_ = 0.024), confirming that 2-point and 3-point shots are very distinct types of shooting motions based on their biomechanical characteristics. Hence, they should not be evaluated together. 

A follow-up ANOVA for the 2-point shooting motion indicated that PRO shooters had greater mean values for elbow angle (F[1,85] = 4.936, *p* = 0.029), shoulder angle (F[1,85] = 5.034, *p* = 0.027), elbow height (F[1,85] = 6.492, *p* = 0.013), basketball height (F[1,85] = 8.981, *p* = 0.004), release angle (F[1,85] = 4.466, *p* = 0.038), and entry angle (F[1,85] = 4.546, *p* = 0.036) when compared to N-PRO shooters. On the other hand, a follow-up ANOVA for 3-point shooting motion revealed that PRO 3-point shooters had greater mean values for hip angle (F[1,85] = 11.018, *p* = 0.001), elbow angle (F[1,85] = 12.590, *p* = < 0.001), basketball height (F[1,85] = 13.044, *p* = < 0.001), release angle (F[1,85] = 16.208, *p* = < 0.001), release height (F[1,85] = 32.278, *p* = < 0.001), heel height (F[1,85] = 22.163, *p* = < 0.001), and maximal trajectory height (F[1,85] = 10.106, *p* = 0.002) when compared to N-PRO shooters. See Table 1 and Table 2 for detailed results. 

**Figure 1:**
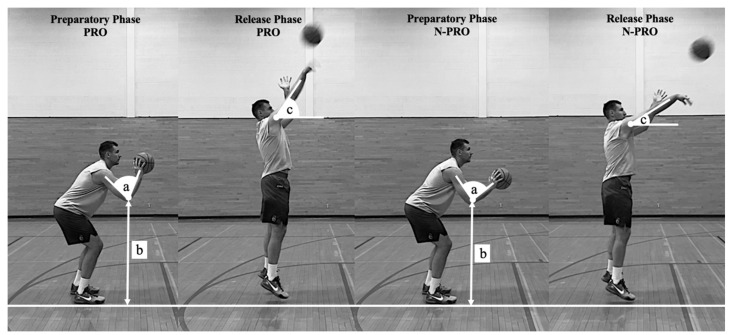
Graphical representation of three key coaching variables discriminating between PRO and N-PRO 2-point shooters. (**a**) elbow angle; (**b**) elbow height; (**c**) release angle.

**Figure 2:**
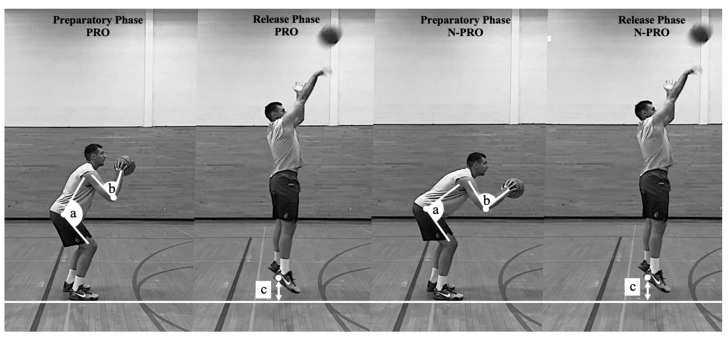
Graphical representation of three key coaching variables discriminating between PRO and N-PRO 3-point shooters. (**a**) hip angle; (**b**) elbow angle; (**c**) heel height.

Three variables with the lowest collinearity (*r* < 0.70) and greatest practical importance were selected to be entered into a full model discriminant analysis for each shooting motion, 2-point and 3-point, separately. See Figure 1 and Figure 2 for detailed graphical representation. A full-model multivariate discriminant function model for the 2-point shooting approach based on elbow angle, elbow height, and release angle variables was statistically significant (Λ = 0.883, X2[3] = 10.405, *p* = 0.015) and capable of correctly classifying shooters based on their proficiency in 62.1% of cases. A multivariate discriminant function model for the 3-point shooting approach based on hip angle, heel height, and elbow angle was also statistically significant (Λ = 0.638, X2[3] = 37.544, *p* = < 0.001) and capable of correctly classifying shooters based on their proficiency in 81.6% of cases. See Table 3 for standardized discriminant function coefficients, percentage of explained variance, and percentage of the total variance. 

## 4. Discussion

Regardless of the type of shooting approach, the findings of the present study indicate significant differences in kinetic and kinematic characteristics between PRO and N-PRO 2-point and 3-point shooters. PRO 2-point shooters demonstrated higher elbow placement caused by greater shoulder and elbow flexion which ultimately elicited an increase in basketball height during the preparatory phase of the shooting motion. The non-significant changes in the hip and knee angle variables for 2-point shooting motion indicate that these adjustments can be primarily attributed to the alterations in the upper-body kinematics. Similar findings regarding the importance of elbow flexion have been previously reported by Yates [5] who found that more successful shooters utilized greater elbow flexion at the start of the jump-shooting motion when compared to poor performers. On the other hand, Syaukani & Yan [17] found that less-experienced shooters tended to flex their shoulder during the preparatory phase of the shooting motion while their experienced counterparts tended to do the opposite and keep the shoulder in a more extended position. Additionally, the overall shooting-shoulder angular displacement was greater for the group of less-experienced shooters resulting in higher positioning of the basketball [17]. While not evaluated by Syaukani & Yan [17], we can assume that the height of the elbow during the initial phase of the shooing motion for less-experienced shooters was greater as a result of the previously indicated kinematic adjustments, which is contradictory to the findings of the present study. The discrepancy in findings may be mainly related to the distance from which jump-shots were attempted, notably smaller sample size, and comparing differences in kinematic parameters of the jump-shooting technique based on the participants’ level of expertise without actually testing for shooting percentage as a measure of shooting proficiency. 

During the preparatory phase of the 3-point shooting motion PRO shooters attained greater elbow flexion, higher basketball height positioning, and less hip flexion. Despite the increase in placement of the basketball, no significant differences in the elbow height and shoulder angle have been found such as seen within PRO 2-point shooters. This may be mainly attributed to the distinct nature of 2-point and 3-point shooting motions caused by an increase in shooting distance [10,18]. Based on the previously mentioned kinematic adjustments regarding the 3-point shooting motion, we can assume that the increase in basketball height during the initial phase was primarily attained by keeping the torso in a more erected position, instead of increasing shoulder flexion and elbow elevation. Previous research has reported that minimizing horizontal sway of the body and maintaining nearly vertical alignment of the trunk during the jump-shooting motion is a movement characteristic of skilled shooters [4]. The mean value of the hip flexion for the PRO 3-point shooters was similar to the one observed in a recently conducted case study analyzing jump-shoot kinematic characteristics of a former collegiate basketball player [19]. Thus, based on the findings of this investigation, alongside appropriate elbow flexion and basketball height, the ability to maintain near-vertical torso positioning and prevent body sway during the preparatory phase of the 3-point shooting motion allowed PRO shooters to acquire superior shooting accuracy. Additionally, a kinematic variable that has not been examined in the present investigation and could have influenced an increase in basketball height is the forearm positioning. Aligning the forearm parallel, or close to parallel, with an imaginary vertical line during the preparatory phase of the shooting motion has been associated with superior shooting performance, for both free-throw and jump-shooting motions [4,6]. Therefore, PRO 3-point shooters may have attained optimal forearm positioning by tucking the elbow in without altering the elbow height which ultimately resulted in an increased basketball height. 

When analyzing trajectory parameters of the 2-point shooting motion, PRO shooters obtained greater release and entry ball angles. Although no significant differences in release and trajectory heights have been detected, a notable increase in release angle could have influenced slight changes in the parabolic trajectory of the ball and caused a small but significant increase in entry ball angle. The direct relationship between release and entry ball angle has been previously reported by Brancazio [20]. The release angle magnitude for 2-point shooting motions obtained in the present study lays within the proposed mid-range jump-shots recommendations; 49–55 degrees [21]. Also, based on the experimental data and theory, it has been suggested that shooting at angles near 52 degrees may allow for optimal release speeds and desirable entry angles [4]. Previous research has also found that with an increase in entry angle the virtual target is enlarged and produces a greater entrance area on the hoop [13,22]. Thus, by making small adjustments in release angle, as a variable that can be manipulated as a part of the shooter’s form, PRO 2-point shooters were capable of achieving a greater entry angle of the ball which ultimately allowed for greater shooting accuracy. 

Greater release angle has remained a prominent characteristic of PRO shooters even when shots were attempted from 3-point shooting distance. Additionally, PRO 3-point shooters were capable of attaining greater heel, release, and trajectory heights. It has been found that greater release angle accomplished by greater flexion in the shoulder joint subsequently caused an increase in release height of the ball [13], which confirms the findings of the present investigation. Despite the slight decrease in release angle magnitude (~2 degrees) influenced by an increase in shooting distance [10,12], the magnitudes of the release angle remained within the previously indicated optimal ranges [4,21]. Bearing in mind that the heel height is a kinematic variable used in this present study as a representative of body vertical displacement, it is believed that by jumping higher PRO 3-point shooters additionally contributed to an increase in release height. Moreover, based on discriminant function analysis findings, heel height was the variable with the greatest influence on discriminating between PRO and N-PRO 3-point shooters. These findings are in the agreement with theoretical computations derived by Brancazio [20] indicating that shooting accuracy is positively related to an increase in release height. Moreover, identical findings regarding the importance of greater height of release have been observed for free-throw shooting performance where with every 0.152 m increase in release height above 1.981 m, shooting accuracy improved by 5% [23]. 

To our knowledge, this is the first investigation focused on examining differences in kinetic characteristics between PRO and N-PRO 2-point and 3-point shooters as well as how they change with implementing different types of shooting approaches. Interestingly, no differences have been found for peak concentric and landing forces, impulse, and rate of force development variables. Struzik et al. [24] found similarity in ground reaction force curve profiles between jump-shooting motion without a ball and maximal countermovement vertical jump without an arm swing. However, the comparison of Struzik et al. [24] findings with the findings of the present investigation is unfeasible as the authors focused on examining mean instead of peak force and power characteristics. On the other hand, when compared to kinetic characteristics for some of the commonly used basketball dunking motions, the magnitudes of peak concentric force, impulse, and rate of force development for 2-point and 3-point shooting approaches were remarkably lower [25,26], which is expected considering the explosive nature of the dunk motion. 

When interpreting the findings of the present study, it is important to note that the testing procedures were performed in an isolated laboratory setting without the opponent’s presence. Additionally, the influence of fatigue was eliminated as a factor that could alter elementary kinetic and kinematic parameters of 2-point and 3-point shooting motions. It has been found that defensive presence caused a player to modify regular shooting technique and respond by using a greater elbow extension, higher ball release angle, lower ball release height, and greater shoulder flexion [27]. Also, the findings regarding the influence of fatigue on kinematic parameters of shooting performance are equivocal [28,29]. Uygyr et al. [28] reported that shooting accuracy and kinematic parameters of free-throw shooting motion were unaffected, while Slawinski et al. [29] found significant changes in hip and shoulder joint flexion in 3-point shooting mechanics as a result of repetitive sprint fatiguing protocols. Considering the nature of the game of basketball as a dynamic sport where the presence of fatigue is unavoidable and where the player with the ball is in close proximity to one or more opponents, future research needs to examine kinetic and kinematic parameters of various types of 2-point and 3-point shooting motions and how they change during live in-game situations. Moreover, future research needs to examine if the observed differences are consistent across different sexes, playing positions, and various levels of basketball competition (e.g., high school, collegiate, professional) as well as to determine their normative ranges.

Although multiple differences in kinematic variables between PRO and N-PRO have been observed, the present study has identified three kinematic variables with the greatest contribution to the successful shooting outcome for each type of shooting motion (2-point and 3-point) that coaches may want to address during regular training sessions. The most critical kinematic variables for optimal 2-point shooting performance were elbow angle, elbow height, and release angle. Instructing athletes to position the elbow higher and increase elbow flexion during the preparatory phase of the shooting motion and increase shoulder flexion at the time point of the ball release to achieve greater release angle may serve as beneficial coaching ques that can help athletes improve their mid-range 2-point shooting performance. On the other hand, due to the different biomechanical requirements influenced by an increase in shooting distance, the three key variables related to optimal 3-point shooting performance were hip angle, elbow angle, and heel height. Therefore, by instructing athletes to increase elbow flexion and keep their torso erected in near-vertical position during the preparatory phase of the shooting motion and jump higher at the time point of the ball release may serve as beneficial coaching cues to help athletes improve their 3-point shooting performance.

## 5. Conclusions

Based on the findings of the present study, the type of shooting approach had no impact on kinetic and kinematic parameters of 2-point and 3-point shooting motions. During the preparatory phase of the shooting motion, PRO 2-point shooters demonstrated higher elbow and basketball height placements, greater flexion in the shoulder and elbow joints while attaining greater release and entry ball angles during the release phase. Conversely, PRO 3-point shooters demonstrated greater elbow flexion, higher basketball placement, and less hip flexion during the preparatory phase while attaining greater heel, release, and trajectory heights during the release phase. When entered into a full-model discriminant function analysis, elbow angle, elbow height, and release angle variables correctly classified PRO from N-PRO 2-point shooters in 62.1% of cases, and hip angle, heel height, and elbow angle variables correctly classified PRO from N-PRO 3-point shooters in 81.6% of cases.

## Figures and Tables

**Table 1:** Mean and standard deviation ($\bar{x}$ ± SD), and coefficient of variation percentage [%] for each variable and shooting approach attempted from 2-point shooting distance.

		**Knee Angle**	**Hip Angle**	**Elbow Angle**	**Ankle Angle**
Stationary					
N-PRO		107.45 ± 11.78 [10.96]	131.85 ± 11.88 [9.01]	63.58 ± 12.25 [19.27]	54.94 ± 8.43 [15.34]
PRO		110.16 ± 11.40 [10.35]	137.92 ± 14.91 [10.81]	58.61 ± 8.99 [15.33]	53.91 ± 5.92 [10.99]
Step-in Left					
N-PRO		109.79 + 8.60 [7.83]	133.84 ± 9.32 [6.97]	65.58 ± 10.76 [16.41]	55.52 ± 6.61 [11.91]
PRO		109.65 ± 11.17 [10.19]	135.88 ± 12.56 [9.24]	60.92 ± 10.03 [16.46]	55.13 ± 5.00 [9.06]
Step-in Right					
N-PRO		106.78 ± 8.08 [7.57]	131.37 ± 8.53 [6.49]	64.79 ± 11.95 [18.44]	55.35 ± 5.81 [10.50]
PRO		108.96 ± 10.95 [10.04]	135.59 ± 11.78 [8.69]	59.23 ± 9.64 [16.27]	54.48 ± 5.33 [9.78]
Total					
N-PRO		108.01 ± 9.51 [8.80]	132.35 ± 9.86 [7.45]	64.65 ± 11.45 [17.71]	55.27 ± 6.89 [12.47]
PRO		109.59 ± 10.89 [9.94]	136.46 ± 12.84 [9.41]	59.58 ± 9.36 [15.71] *	54.51 ± 5.31 [9.74]
		**Shoulder Angle**	**Elbow Height**	**Basketball Height**	**Release Angle**
Stationary					
N-PRO		75.73 ± 28.01 [36.98]	0.65 ± 0.11 [16.50]	0.85 ± 0.10 [12.01]	49.86 ± 11.11 [22.28]
PRO		85.71 ± 16.31 [19.03]	0.70 ± 0.09 [12.37]	0.90 ± 0.08 [9.32]	54.08 ± 7.48 [13.82]
Step-in Left					
N-PRO		74.38 ± 27.63 [37.14]	0.66 ± 0.08 [12.59]	0.85 ± 0.08 [9.52]	49.58 ± 11.60 [23.39]
PRO		85.50 ± 15.75 [18.42]	0.70 ± 0.08 [11.98]	0.90 ± 0.08 [8.68]	54.38 ± 8.40 [15.44]
Step-in Right					
N-PRO		74.73 ± 28.90 [38.68]	0.65 ± 0.09 [14.01]	0.85 ± 0.09 [11.10]	49.86 ± 11.42 [22.91]
PRO		86.76 ± 14.12 [16.27]	0.70 ± 0.08 [10.91]	0.90 ± 0.07 [7.33]	54.26 ± 8.41 [15.49]
Total					
N-PRO		74.95 ± 27.58 [36.80]	0.65 ± 0.09 [13.85]	0.85 ± 0.09 [10.59]	49.77 ± 11.13 [22.36]
PRO		85.99 ± 15.01 [17.46] *	0.70 ± 0.08 [11.43] *	0.90 ± 0.07 [7.78] *	54.24 ± 7.89 [14.55] *
		**Release Height**	**Heel Height**	**Trajectory Height**	**Entry Ball Angle**
Stationary					
N-PRO		1.35 ± 0.11 [8.35]	20.28 ± 8.22 [40.55]	391.18 ± 26.35 [6.74]	43.28 ± 2.88 [6.55]
PRO		1.39 ± 0.05 [3.72]	19.25 ± 3.92 [20.39]	395.9 ± 17.39 [4.39]	44.59 ± 1.40 [3.15]
Step-in Left					
N-PRO		1.39 ± 0.11 [8.15]	25.58 ± 7.15 [27.96]	393.83 ± 21.16 [5.37]	43.69 ± 2.54 [5.81]
PRO		1.40 ± 0.07 [4.82]	21.68 ± 4.15 [19.16]	397.94 ± 15.89 [3.99]	44.44 ± 1.16 [2.60]
Step-in Right					
N-PRO		1.39 ± 0.12 [8.33]	24.61 ± 7.07 [28.72]	393.60 ± 23.35 [5.93]	43.52 ± 2.69 [6.18]
PRO		1.40 ± 0.07 [4.96]	21.68 ± 3.31 [15.27]	399.58 ± 16.65 [4.17]	44.4 ± 1.16 [2.62]
Total					
N-PRO		1.37 ± 0.11 [8.03]	23.48 ± 7.69 [32.75]	392.87 ± 23.23 [5.91]	43.49 ± 2.62 [6.02]
PRO		1.39 ± 0.06 [4.32]	20.87 ± 3.83 [18.35]	397.81 ± 16.28 [4.09]	44.48 ± 1.21 [2.71] *
		**Peak Concentric Force**	**Peak Landing Force**	**Impulse**	**RFD**
Stationary					
N-PRO		1679.86 ± 385.44 [22.94]	1652.63 ± 577.96 [34.97]	154.35 ± 51.72 [33.51]	4386.45 ± 2548.93 [58.11]
PRO		1705.99 ± 441.24 [25.86]	1811.18 ± 510.10 [28.16]	147.68 ± 41.45 [28.07]	4430.81 ± 3501.55 [79.03]
Step-in Left					
N-PRO		1942.45 ± 467.74 [24.08]	2080.41 ± 753.69 [36.23]	181.25 ± 56.85 [31.36]	6269.54 ± 3732.51 [59.53]
PRO		1806.77 ± 464.63 [25.72]	2028.27 ± 502.12 [24.76]	158.72 ± 40.04 [25.23]	5550.85 ± 4169.06 [75.11]
Step-in Right					
N-PRO		1950.75 ± 513.43 [26.32]	1927.66 ± 593.16 [30.77]	176.16 ± 52.49 [29.81]	6500.51 ± 3672.60 [56.51]
PRO		1804.38 ± 472.48 [26.19]	1981.04 ± 433.62 [21.89]	153.31 ± 41.61 [27.14]	5739.81 ± 4020.42 [70.04]
Total					
N-PRO		1857.68 ± 466.41 [25.11]	1886.90 ± 657.41 [34.84]	170.58 ± 53.89 [31.59]	5718.83 ± 3426.38 [59.91]
PRO		1772.38 ± 449.01 [25.33]	1940.16 ± 479.67 [24.67]	153.23 ± 40.21 [26.24]	5240.49 ± 4202.68 [67.35]

Note: * significantly different compared to N-PRO “Total” (*p* < 0.05). PRO = participants making ≥50% of their 2-point and shots. PRO = participants making <50% of their 2-point shots.

**Table 2:** Mean and standard deviation ($\bar{x}$ ± SD) and coefficient of variation percentage [%] for each variable and shooting approach attempted from 3-point shooting distance.

		**Knee Angle**	**Hip Angle**	**Elbow Angle**	**Ankle Angle**
Stationary					
N-PRO		104.2 ± 10.53 [10.10]	127.58 ± 13.43 [10.52]	65.89 ± 11.48 [17.43]	50.89 ± 5.04 [9.91]
PRO		103.49 ± 11.06 [10.69]	133.69 ± 8.76 [6.56]	57.38 ± 9.59 [16.72]	52.18 ± 6.23 [12.03]
Step-in Left					
N-PRO		104.68 ± 10.24 [9.78]	126.89 ± 11.18 [8.81]	66.98 ± 10.31 [15.41]	52.20 ± 6.39 [12.24]
PRO		105.66 ± 10.71 [10.14]	135.92 ± 8.56 [6.31]	59.49 ± 9.54 [16.04]	52.25 ± 4.92 [9.42]
Step-in Right					
N-PRO		103.16 ± 9.98 [9.67]	126.75 ± 11.65 [9.19]	65.85 ± 11.27 [17.11]	51.89 ± 4.98 [9.60]
PRO		103.66 ± 11.48 [11.08]	134.15 ± 8.71 [6.49]	58.42 ± 9.75 [16.68]	51.53 ± 5.52 [10.71]
Total					
N-PRO		104.01 ± 10.05 [9.67]	127.07 ± 11.87 [9.34]	66.24 ± 10.81 [16.32]	51.66 ± 5.42 [10.49]
PRO		104.27 ± 10.84 [10.40]	134.59 ± 8.51 [6.32] *	58.43 ± 9.41 [16.11] *	51.99 ± 5.46 [10.51]
		**Shoulder Angle**	**Elbow Height**	**Basketball Height**	**Release Angle**
Stationary					
N-PRO		72.59 ± 25.75 [35.48]	0.62 ± 0.09 [14.72]	0.81 ± 0.09 [10.89]	44.48 ± 11.99 [26.97]
PRO		71.98 ± 27.15 [37.73]	0.66 ± 0.09 [13.96]	0.86 ± 0.11 [12.19]	53.06 ± 6.11 [11.52]
Step-in Left					
N-PRO		69.22 ± 28.22 [40.77]	0.62 ± 0.08 [14.14]	0.79 ± 0.09 [11.45]	44.51 ± 11.77 [26.46]
PRO		71.61 ± 27.91 [38.97]	0.66 ± 0.09 [12.86]	0.87 ± 0.09 [11.39]	52.78 ± 6.27 [12.74]
Step-in Right					
N-PRO		69.41 ± 27.43 [39.52]	0.66 ± 0.21 [31.63]	0.80 ± 0.08 [10.33]	44.27 ± 11.92 [26.94]
PRO		72.86 ± 28.66 [39.32]	0.66 ± 0.09 [13.20]	0.87 ± 0.09 [11.24]	52.55 ± 6.67 [12.67]
Total					
N-PRO		70.40 ± 26.62 [37.81]	0.63 ± 0.14 [21.87]	0.79 ± 0.09 [10.71]	44.41 ± 11.64 [26.22]
PRO		72.15 ± 27.17 [37.66]	0.66 ± 0.09 [12.99]	0.87 ± 0.09 [11.31] *	52.81 ± 6.34 [12.01] *
		**Release Height**	**Heel Height**	**Trajectory Height**	**Entry Ball Angle**
Stationary					
N-PRO		1.33 ± 0.10 [7.52]	22.7 ± 4.07 [17.92]	426.07 ± 26.33 [6.18]	44.05 ± 2.43 [5.52]
PRO		1.47 ± 0.10 [6.80]	28.93 ± 4.96 [17.16]	443.35 ± 17.71 [3.99]	45.33 ± 2.81 [6.19]
Step-in Left					
N-PRO		1.36 ± 0.11 [8.01]	25.42 ± 5.36 [21.08]	430.04 ± 26.06 [6.06]	44.66 ± 2.91 [6.51]
PRO		1.481 ± 0.11 [7.20]	30.67 ± 6.63 [21.64]	442.93 ± 13.55 [3.06]	45.28 ± 2.17 [4.78]
Step-in Right					
N-PRO		1.36 ± 0.11 [8.24]	24.81 ± 4.86 [19.56]	429.72 ± 27.11 [6.31]	43.88 ± 2.75 [6.27]
PRO		1.48 ± 0.10 [6.59]	29.65 ± 6.41 [21.59]	443.85 ± 12.04 [2.71]	44.89 ± 1.79 [3.99]
Total					
N-PRO		1.35 ± 0.11 [7.82]	24.32 ± 4.83 [19.87]	428.61 ± 25.99 [6.06]	44.19 ± 2.67 [6.03]
PRO		1.47 ± 0.10 [6.71] *	29.75 ± 5.93 [19.93] *	443.38 ± 14.24 [3.21] *	45.16 ± 2.24 [4.96]
		**Peak Concentric Force**	**Peak Landing Force**	**Impulse**	**RFD**
Stationary					
N-PRO		1911.57 ± 378.61 [19.81]	2059.76 ± 554.91 [26.94]	183.56 ± 46.09 [25.11]	5537.22 ± 3020.48 [54.55]
PRO		1922.08 ± 346.32 [18.01]	2309.21 ± 546.21 [23.65]	187.16 ± 42.61 [22.77]	4884.15 ± 2195.22 [44.95]
Step-in Left					
N-PRO		2059.74 ± 499.73 [24.26]	2208.58 ± 568.67 [25.75]	194.02 ± 45.99 [23.71]	7206.91 ± 4259.81 [59.11]
PRO		2021.01 ± 395.37 [19.57]	2336.67 ± 521.63 [22.32]	189.99 ± 43.41 [22.84]	6499.17 ± 3690.78 [56.79]
Step-in Right					
N-PRO		2078.01 ± 509.92 [24.53]	2165.18 ± 588.68 [27.19]	187.56 ± 37.29 [19.88]	7713.82 ± 4690.67 [60.81]
PRO		2005.46 ± 380.65 [18.98]	2338.91 ± 518.03 [22.15]	188.34 ± 42.83 [22.74]	6277.75 ± 3568.82 [56.84]
Total					
N-PRO		2016.44 ± 462.71 [22.95]	2144.50 ± 562.19 [26.22]	188.38 ± 42.61 [22.62]	6819.32 ± 4075.29 [59.76]
PRO		1982.84 ± 367.37 [18.53]	2328.26 ± 514.84 [22.11]	188.49 ± 41.82 [22.19]	5887.02 ± 3220.25 [54.70]

Note: * significantly different compared to N-PRO “Total” (*p* < 0.05). PRO = participants making ≥40% of their 3-point and shots. PRO = participants making <40% of their 3-point shots.

**Table 3:** Standardized discriminant function coefficients, percentage of explained variance, and percentage of total variance for 2-point and 3-point shooting motions.

Dependent Variables	Standardized Coefficients	Percentage of Explained Variance	Percentage of Total Variance
2-point			
Elbow Angle	−0.668	43.69	27.13
Elbow Height	0.792	51.80	32.17
Release Angle	−0.069	4.51	2.80
		100	62.1
3-point			
Hip Angle	0.458	26.32	21.48
Elbow Angle	−0.523	30.06	24.53
Heel Height	0.759	43.62	35.59
		100	81.6

## Data Availability

The data presented in this study are available on request from the corresponding author. The data are not publicly available due to IRB-imposed restrictions.
